# How self-leadership promotes job crafting: Based on the perspective of self-determination theory

**DOI:** 10.3389/fpsyg.2023.1079196

**Published:** 2023-03-02

**Authors:** Geng Liu, Huimin Peng, Hao Wen

**Affiliations:** ^1^School of Economics and Management, Jiangxi Agricultural University, Nanchang, China; ^2^School of Business Administration, Zhongnan University of Economics and Law, Wuhan, China

**Keywords:** self-leadership, autonomous motivation, job crafting, leader empowering behavior, self-determination theory

## Abstract

According to self-determination theory, the present study develops a moderated mediation model to investigate how and when self-leadership promotes employees’ job crafting, emphasizing the mediating effect of autonomous motivation and the moderating effect of leader empowering behavior. We analyze and test the hypotheses based on 269 valid three-wave data from employees. The findings show that self-leadership has a significantly positive impact on job crafting, and a positive indirect effect on job crafting *via* autonomous motivation. Furthermore, leader empowering behavior not only enhances the positive impact of self-leadership on autonomous motivation, but also positively moderates the mediating effect of autonomous motivation in the relationship between self-leadership and job crafting. Practically, our study provides insights into how to promote job crafting. We also propose limitations and directions for future research.

## 1. Introduction

With the development of the global economy, the business environment becomes more and more competitive, uncertain, and complex. Against this background, it is difficult for traditional top-down job design to meet the needs of organizational development and employees’ demands for autonomous and personalized work. Job crafting, which refers to self-initiated behaviors that help employees shape and redefine their jobs (Wrzesniewski and Dutton, 2001), has become an important topic of common concern for academics and managers. Previous studies have shown that job crafting can bring various individual-level and organizational-level benefits (Rudolph et al., 2017), including increased job engagement (Petrou et al., 2012) and job satisfaction (Villajos et al., 2019); improved well-being (Tims et al., 2013) and work meaningfulness (Tims et al., 2016); as well as enhanced organizational commitment and organizational attachment (Wang et al., 2018).

Given that job crafting has been shown to have a substantial effect on employees and better serve organizational goals in a changing environment (Wang et al., 2017), there is increasing interest in identifying factors that enhance job crafting, mainly focusing on personality characteristics (Bakker et al., 2012), job characteristics (Niessen et al., 2016) and leadership styles (Zhang and Parker, 2019). Especially, recent studies have indicated that leaders are the key factors in the organizational context, whose leadership styles play a crucial role in shaping job crafting (e.g., transformational leadership, Wang et al., 2017; servant leadership, Khan et al., 2021). This series of studies, which focused on top-down influence processes and highlighted the role of leaders’ leadership styles, has expanded our understanding of the influence of leadership on employees’ job crafting. However, it is a pity that the existing literature has almost neglected the impact of employee-oriented self-leadership on job crafting. Self-leadership, an internal self-influence process, has gradually attracted some scholars’ attention (Harari et al., 2021; Knotts et al., 2022). Unlike general leadership styles, which highlight the influence of leaders’ symbolic behaviors on employees, self-leadership aims at achieving self-guidance and self-motivation *via* using a specific set of behavioral and cognitive strategies (Houghton and Neck, 2002; Neck and Houghton, 2006). Besides, self-leadership has more positive values for individuals in the era of digital technology and information (Knotts et al., 2022). Therefore, we contend that it has great theoretical significance and practical implications to explore the relationship between self-leadership and job crafting.

This study draws from self-determination theory (Deci and Ryan, 2002) to put forward a moderated mediation model that reveals how and when self-leadership influences employees’ job crafting. According to self-determination theory, the context that is good for the satisfaction of individuals’ basic psychological needs for autonomy, competence, and relatedness can generate autonomous motivation, which in turn promotes employees’ proactive behaviors (Deci and Ryan, 2000; Deci et al., 2017). Meanwhile, external contextual factors usually influence the formation of autonomous motivation by satisfying the basic psychological needs of individuals (Deci and Ryan, 2000). Relevant studies have shown that self-leaders experience more self-determination and a sense of ownership over their work (Neck and Houghton, 2006; Stewart et al., 2011), which creates a supportive context that is conducive to the fulfillment of basic psychological needs. Moreover, autonomous motivation, as a kind of behavioral motivation, refers to the psychological drive of individuals to take actions because of personal will and choice (Deci and Ryan, 2000), and has been considered to result in positive work behaviors and consequences (Gagné and Deci, 2005). Therefore, we propose that self-leadership can promote employees’ basic psychological needs satisfaction, so that employees’ autonomous motivation will be enhanced, resulting in some positive employee behaviors such as increased job crafting.

Furthermore, self-determination theory points out that the impact of individual factors on employees’ motivations and behaviors is influenced by external contextual factors (Ryan and Deci, 2000). Gagné and Deci (2005) also proposed that when using self-determination theory to explore individual motivation, the interactive impact of the social context and individual differences on motivation should be considered. Indeed, the leader is a very crucial person in the social context and has a significant effect on strengthening or weakening the employee’s motivation to behave proactively (Parker and Wu, 2014; Fuller et al., 2015). Leader empowering behavior is defined as leaders’ top–down assignment of responsibilities to employees, information-sharing with employees, and granting employees more decision-making power to engage in work behaviors (Xiang et al., 2021). As an important contextual factor, leader empowering behavior is good for fostering a supportive environment full of autonomy, thus improving the effectiveness of self-leadership (Namasivayam et al., 2014). Therefore, this study introduces leader empowering behavior as a contingent factor to explore the boundary condition in the process of shaping job crafting.

**Figure 1:**
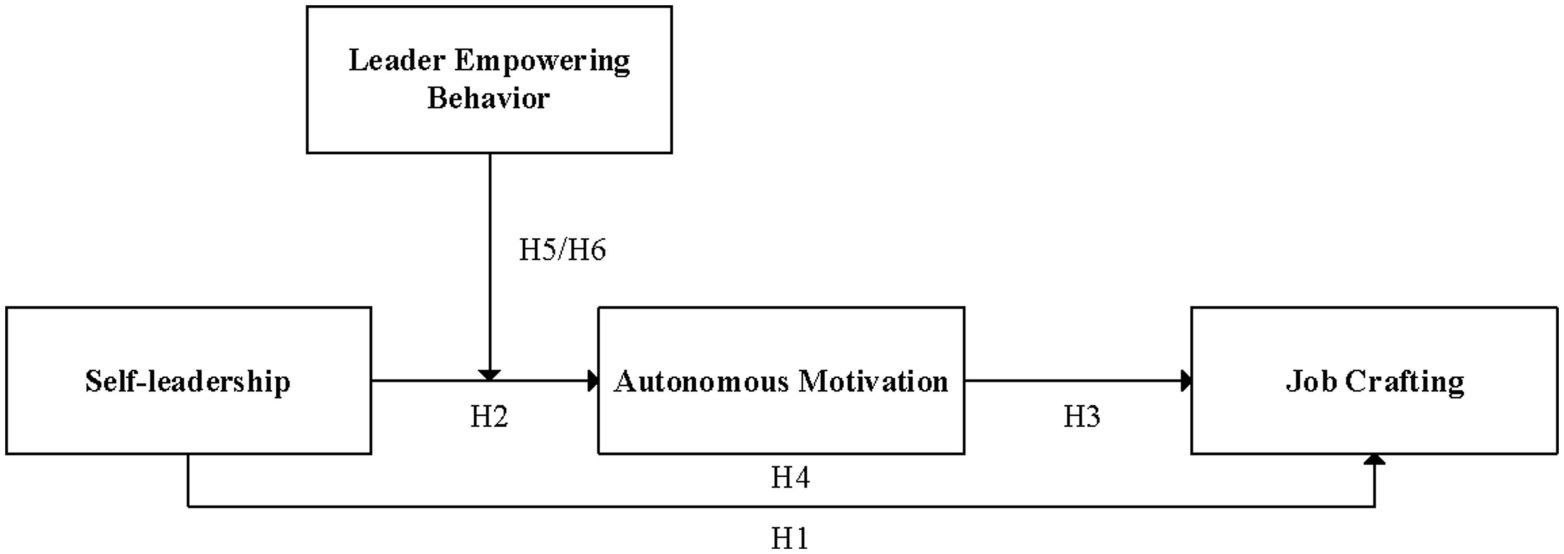
Theoretical model.

Our study aims to contribute to the literature in the following three ways. First of all, our study extends the literature on antecedents of job crafting from the self-influence perspective by examining the positive impact of self-leadership on job crafting. Specifically, this study adds self-leadership, the process of leading from the inside out (Knotts et al., 2022), as an antecedent of job crafting, thus providing a supplement to the limited antecedent research about the effect of individual differences on job crafting. It also responds to the call for studies to investigate the process of associating leadership with job crafting (Berg et al., 2013). Second, based on self-determination theory, our study clarifies how self-leadership affects job crafting by uncovering the mediating effect of autonomous motivation. This deepens our understanding of the internal motivational mechanism by which self-leadership influences job crafting from the perspective of motivation. Finally, our study enriches the boundary conditions in the process of shaping job crafting by identifying leader empowering behavior as a critical contingent factor on the relationship between self-leadership and job crafting from the work contextual factor. To sum up, the theoretical model for this study is presented in Figure 1.

## 2. Theory and hypotheses

Job crafting is a bottom-up, individualized, and spontaneous behavior (Wrzesniewski and Dutton, 2001; Rudolph et al., 2017). Since job crafting is an effective measure to improve employees’ well-being and work motivation, especially in the rapidly changing work environment (Kim and Beehr, 2018), some scholars have been interested in exploring factors promoting employees’ job crafting, such as job characteristics (Niessen et al., 2016) and leadership styles (Zhang and Parker, 2019). Previous studies have declared that leadership plays a vital role in fostering employees’ job crafting (Park and Park, 2021). On the one hand, several studies have indicated that positive leadership styles can facilitate employees’ job crafting mainly by improving job autonomy (Sekiguchi et al., 2017), providing job resources, and creating a work environment full of trust. First of all, some leadership styles which emphasize leaders’ supportive and empowering characteristics can improve employees’ job crafting (Wrzesniewski and Dutton, 2001), such as servant leadership (Khan et al., 2021) and empowering leadership (Kim and Beehr, 2018). Next, leaders who tend to be open and appreciate employees will give employees opportunities to proactively craft their jobs (Park and Park, 2021), such as transformational leadership (Wang et al., 2017) and humble leadership (Ding et al., 2020). At last, from a relational perspective, some leadership styles help maintain high-quality relationships with employees, which can enhance employees’ motivation to engage in job crafting, such as leader-member exchange (Lee, 2020) and authentic leadership (Luu, 2020). On the other hand, a few studies have found that negative leadership styles may hinder employees to craft their jobs. For example, authoritarian leaders monopolize information, devalue employees’ abilities and ignore subordinates’ suggestions and contributions, which will weaken the psychological empowerment and self-efficacy of employees, thus inhibiting their job crafting (Tuan, 2018).

Although existing studies have expanded our knowledge of the relationship between leadership styles and employees’ job crafting, these studies mainly view leaders or supervisors as the initiators of leadership behaviors (Knotts et al., 2022) and focus on investigating how leaders or supervisors affect employees from the top-down (Stewart et al., 2011). In recent years, scholars take an alternative perspective to pay attention to how individuals manage and lead themselves (Stewart et al., 2011). Self-leadership has been proven to promote productive cognition, attitudes, and behaviors of employees (Harari et al., 2021). Meanwhile, with the popularization of digital technology and the rapid change of work environment, the effectiveness and necessity of self-leadership are increasingly prominent (Knotts et al., 2022). However, few empirical studies have adequately examined the relationship between self-leadership and employees’ job crafting.

### 2.1. Self-leadership and job crafting

Self-leadership refers to “a self-influence process through which individuals achieve the self-direction and self-motivation necessary to perform” (Neck and Houghton, 2006, p. 271). Established studies have revealed that self-leaders are confident and have a sense of ownership because of the control over their work (Stewart et al., 2011). Individual self-leadership is usually seen as a valuable capability because it has been linked to a couple of positive work-related outcomes, such as greater job satisfaction (Neck and Manz, 1996), higher self-efficacy (Prussia et al., 1998), and greater career success (Raabe et al., 2007). Rudolph et al. (2017) have pointed out that job autonomy and self-efficacy have a positive effect on job crafting. Thus, we contend that self-led employees have enough autonomy and control over their tasks to perceive that they have the opportunity to transform and reshape their jobs, thus leading to increased job crafting. Moreover, self-leadership can stimulate several employees’ positive work behaviors, such as innovation behavior (Carmeli et al., 2006) and organizational citizenship behavior (Jensen and Raver, 2012). Therefore, we speculate that job crafting, a specific form of proactive work behavior (Khan et al., 2021), may be positively affected by self-leadership.

Furthermore, self-led employees can use a series of specific behavioral and cognitive strategies to control their behaviors, subsequently enhancing their productivity and proactive behaviors. First, behavior-focused strategies include some specific behaviors, such as self-observation, self-goal setting, and self-reward. Employees may improve their self-awareness and enhance self-directed efforts in performing essential but perhaps unpleasant tasks by the implementation of behavior-focused strategies (Neck and Houghton, 2006). And employees can adopt behavior-focused strategies to determine their long-term goals and guide their behavior toward the desired goals. This makes employees be willing to take the initiative to increase work content and adjust methods to achieve goals, which is conducive to the formation of job crafting. Second, natural reward strategies focus on the enjoyable aspects and the positive experience of a given task or activity, which is beneficial to meet individuals’ needs at different levels (Carmeli et al., 2006). When faced with a specific task, employees will motivate themselves to efficiently complete their work by constructing pleasant attributes, which is conducive to enhancing their self-efficacy and making employees redesign their work to obtain the desired results (Prussia et al., 1998). Finally, constructive thought pattern strategies mean that individuals develop constructive thought patterns in three ways, such as evaluating positive beliefs, using mental imagery, and increasing positive self-talk (Neck and Manz, 1996). Some studies have shown that the use of these strategies can help employees improve their self-efficacy, develop positive thinking and behavior, and promote the achievement of desired behavioral outcomes (Ho and Nesbit, 2014). Therefore, we speculate that the three self-leadership strategies are good for enhancing employees’ self-awareness, self-motivation, and intrinsic motivation, which can positively influence employees’ positive work behaviors, such as job crafting. Thus, we present the following hypothesis:

*Hypothesis 1*: Self-leadership is positively related to job crafting.

### 2.2. The mediating role of autonomous motivation

Autonomous motivation is defined as the full approval of one’s own activities or behaviors that are in accordance with their goals, needs, interests, and values (Weinstein and Ryan, 2010), and it describes the psychological drive of an individual to act out of willingness or personal choice (Deci and Ryan, 2000). According to self-determination theory, supportive contextual factors that satisfy the three basic psychological needs are important ways to promote individuals’ autonomous motivation (Ryan and Deci, 2000; Deci et al., 2017). The higher the level of satisfaction with these basic needs, the greater the level of autonomous motivation perceived by individuals (Gagné and Deci, 2005). Self-leadership refers to the process in which employees actively influence and regulate their psychology and behavior to achieve the expected goals (Manz, 1986). In the process of self-leadership, employees can plan their work properly through goal setting, self-observation, and self-reward (Houghton and Neck, 2002; Harari et al., 2021), which is good for creating a supportive circumstance. Thus, we infer that self-leadership may stimulate employees’ autonomous motivation.

First of all, employees who implement self-leadership can set goals on the basis of their own wishes, flexibly adjust the work content, and even can choose how to work according to their preferences. Therefore, during the self-leadership process, employees experience a higher sense of control and self-determination, thus satisfying the need for autonomy. Secondly, self-led employees feel that their behaviors are valuable and have a high sense of work meaning and self-efficacy (Manz, 1986; Prussia et al., 1998). Meanwhile, the use of self-leadership strategies enables employees to have greater confidence in their actions, thus improving their self-efficacy and self-worth. In short, self-leadership heightens the fulfillment of employees’ needs for competence by identifying and improving their actual capacity and ability. Finally, in the process of self-leadership, employees perceive respect, trust, and support from their supervisors and colleagues. This makes employees a strong sense of organizational identification and belonging, which meets their needs for relatedness. Combined with the above analysis, self-leadership is beneficial to motivate employees’ autonomous motivation. Accordingly, the following hypothesis is proposed:

*Hypothesis 2*: Self-leadership is positively related to autonomous motivation.

Further, when employees’ three basic psychological needs (i.e., autonomy, competence, and relatedness) are satisfied, they activate their own autonomous motivation, which may promote job crafting. First, existing studies show that higher autonomous motivation means that employees are more proactive and have a greater sense of autonomy at work (Deci and Ryan, 2000). Job autonomy is an important job characteristic that directly affects job crafting (Wrzesniewski and Dutton, 2001). Accordingly, we contend that autonomous motivation can facilitate employees’ job crafting. Second, autonomous motivation implies that employees are more willing to engage in their work because they internalize the work value and meaning as personal values and beliefs, and genuinely enjoy their work (De Cooman et al., 2013). Finally, autonomous motivation can provide stronger motivation and energy for employees’ actions (Ryan and Deci, 2000), thereby promoting employees to engage in more proactive behaviors. As a proactive organizational behavior, employees’ intrinsic motivation is the direct motivation of job crafting and can play a leading role (Kim et al., 2013). Therefore, employees with higher autonomous motivation are more likely to engage in job crafting. Accordingly, we propose the following hypothesis:

*Hypothesis 3*: Autonomous motivation is positively related to job crafting.

Self-determination theory proposes that individuals have the motivation to satisfy the three basic needs for autonomy, competence, and relatedness, and the meet of them can improve their autonomous motivation and have a positive impact on their behaviors (Deci and Ryan, 2000, 2002). Therefore, employees’ autonomous motivation is a crucial internal mechanism in the process of contextual factors affecting employees’ proactive behaviors. We speculate that the impact of self-leadership on job crafting is mediated by employees’ autonomous motivation. Specifically, self-leadership can activate employees’ intrinsic work motivation, make them feel the meaningfulness and mission of work, and then promote them to participate in proactive behaviors that benefit the organization and individuals (Park et al., 2016). Therefore, when the basic psychological needs of self-led employees are met, they will internalize their work as personal values and integrate them into their autonomous motivation, thus promoting their job crafting. In conclusion, we propose the following hypothesis:

*Hypothesis 4*: Autonomous motivation mediates the relationship between self-leadership and job crafting.

### 2.3. The moderating role of leader empowering behavior

Drawing on the self-determination theory, the influence of individual factors on employees’ motivation and behavior is affected by external contextual factors (Ryan and Deci, 2000). Employees are affected by various factors in their organization, and leadership is an important external contextual factor. Leader empowering behavior reflects an active communication and interaction between leaders and employees (Kim and Beehr, 2018). It enhances employees’ internal motivation and work initiative by empowering employees, promoting employees’ development, and encouraging employees to participate in decision-making and other behaviors, so that employees can have more positive attitudes and behaviors under self-leadership (Thomas and Velthouse, 1990). In addition, the self-leadership model points out that self-leadership is a process of resource depletion, which will consume employees’ own resources (Stewart et al., 2019). The external resources support and the internal forces of employees help to provide sufficient resource supply to solve the short-term resource depletion problem in the process of self-leadership and make self-leadership sustainable (Stewart et al., 2019). As an external resource, leader empowering behavior can support and replenish resources, so that there are sufficient resources to continue and develop self-leadership. Strong resource support strengthens the effectiveness of self-leadership, and then generates stronger autonomous motivation. Therefore, we posit that leader empowering behavior may positively moderate the positive influence of self-leadership on autonomous motivation.

Specifically, high leader empowering behavior gives employees more job autonomy, decision-making power and opportunities to share information (Cheong et al., 2016). When self-led employees are under the situation of high leader empowering behavior, they will get more disposable resources and a higher sense of control, self-efficacy and belonging. Thus, employees’ basic psychological needs are met, and their autonomous motivation will be greatly enhanced. On the contrary, when employees are under the situation of low leader empowering behavior, they will not have enough power to make decisions and exert their own ideas, and get less trust and support from leaders. At this time, they will doubt their work and their own values, and their confidence and autonomy will be weakened. Thus, the effectiveness of self-leadership is weakened and self-leadership is difficult to sustain, thereby weakening the positive effect of self-leadership on autonomous motivation. It can be seen that different levels of leader empowering behavior and employee self-leadership have different degrees of interaction, and then bring different effects on autonomous motivation. Taking these considerations together, we propose the following hypothesis:

*Hypothesis 5*: Leader empowering behavior moderates the relationship between self-leadership and autonomous motivation such that the relationship is stronger when leader empowering behavior is high.

According to the self-determination theory, whether the external contextual factors can facilitate the formation of individual positive attitudes and behaviors depends on whether the individual can effectively interact with the external context (Deci et al., 1989; Ryan and Deci, 2000). When self-led employees are under the situation of high leader empowering behavior, their psychological needs for autonomy, competence and relatedness will be more satisfied, thus enhancing their autonomous motivation and subsequently promoting job crafting. Accordingly, combined with the above discussion, we further propose that the mediating effect of autonomous motivation between self-leadership and job crafting is moderated by leader empowering behavior. Specifically, when employees are under the situation of high leader empowering behavior, their autonomous motivation is more likely to be enhanced by self-leadership, thus promoting job crafting. Conversely, when employees are under the situation of low leader empowering behavior, the influence of self-leadership on their autonomous motivation is weakened, thus reducing job crafting. Therefore, taking hypothesis 4 and hypothesis 5 together, we propose the following hypothesis:

*Hypothesis 6*: Leader empowering behavior moderates the mediation effect of autonomous motivation on the relationship between self-leadership and job crafting, such that the mediation effect is stronger when leader empowering behavior is high.

## 3. Materials and methods

### 3.1. Sample and procedures

To avoid the contextual constraints of industries and generalize the research findings, we collected data from ten Chinese enterprises, such as retailing and real estate. In order to reduce common method variance, we designed and implemented a three-wave data collection, 2 weeks apart. In all surveys, an instruction accompanying the questionnaire showed that the data would be used only for academic research, and the anonymity and confidentiality of responses were guaranteed. At Time 1, each employee was invited to answer his/her demographic variables, and evaluate self-leadership and leader empowering behavior. In this stage, 350 questionnaires were sent out and 326 employees participated in the survey. At Time 2, we invited employees who participated in the Time 1 survey wave to fill out an autonomous motivation scale after 2 weeks. In this stage, 326 questionnaires were sent out and 298 employees participated in the survey. After another 2 weeks, at Time 3, employees were invited to measure their job crafting. In this stage, 286 questionnaires were eventually returned.

We eliminated some invalid questionnaires and finally obtained 269 valid three-wave questionnaires, with an effective response rate of 76.86%. Among the final 269 participants, 45.7% were male and 54.3% were female. In terms of age, the participants were mainly aged 31–40 years old (accounting for 49.1%), followed by 21–30 years old (accounting for 40.1%). In terms of educational background, 16.7% had a college degree or below and 83.3% had a bachelor’s degree or above. The organizational tenure of the participants was mainly 4–6 years (accounting for 33.5%), followed by 7–10 years (accounting for 29.7%). And in terms of organization type, respondents were mainly from state-owned enterprises (accounting for 42.4%) and private enterprises (accounting for 40.9%).

### 3.2. Measurement

The measures of our key variables were originally described in English. In order to guarantee the effectiveness and comprehensibility of the scales in the context of Chinese culture, we followed a standard translation and back-translation procedure (Brislin, 1970) to translate all English scales into Chinese. Moreover, each measure was rated on a five-point Likert scale ranging from 1 (totally disagree) to 5 (totally agree).

#### 3.2.1. Self-leadership

Self-leadership was measured using a 9-item scale developed by Houghton et al. (2012). Sample items are “I establish specific goals for my own performance” and “When I have successfully completed a task, I often reward myself with something I like.” Cronbach’s alpha for this scale in this study was 0.886.

#### 3.2.2. Autonomous motivation

Learning from the study of De Cooman et al. (2013), autonomous motivation was measured using the subscales of the Motivation at Work Scale developed by Gagné et al. (2010), which contained 6 items. Three of them measured intrinsic motivation, such as “Because I enjoy this work very much.” Another three items measured identified regulation, such as “Because this job fulfills my career plans.” Cronbach’s alpha for this scale in this study was 0.820.

#### 3.2.3. Job crafting

Job crafting was measured using a 5-item scale developed by Slemp and Vella-Brodrick (2013). Sample items are “I will introduce new approaches to improve my work” and “I will change the scope or types of tasks that I complete at work.” Cronbach’s alpha for this scale in this study was 0.909.

#### 3.2.4. Leader empowering behavior

Leader empowering behavior was measured using a 17-item six-dimensional scale developed by Konczak et al. (2000). Sample items are “My manager gives me the authority to make changes necessary to improve things” and “My manager shares information that I need to ensure high quality results.” Cronbach’s alpha for this scale in this study was 0.840.

#### 3.2.5. Control variables

We included employees’ gender, age, education, and organizational tenure as control variables, as they have been found to be significantly related to job crafting (De Cooman et al., 2013; Solberg and Wong, 2016; Rudolph et al., 2017). Gender was coded as 0 = male and 1 = female. Education was coded as follows: 1 = junior high school and below, 2 = high school, 3 = junior college, 4 = bachelor, 5 = master, and 6 = doctor. Organizational tenure was coded as follows: 1 = under 1 year, 2 = 1–3 years, 3 = 4–6 years, 4 = 7–10 years, 5 = 11 years or above.

## 4. Analysis and results

### 4.1. Common method variance

Because the data of focal variables were obtained from a single source, there might be common method variance (Podsakoff et al., 2003). We used two methods to examine the possible common method deviation. First, we conducted the Harman single factor test by SPSS 26 (Podsakoff and Organ, 1986) and the results indicated that the variance explained by the first factor was 17.91%, lower than 40% (Podsakoff and Organ, 1986). Thus, the present study had no serious common method variance. Then, after confirmatory factor analysis, a common method factor was added to the four-factor model. The results showed that the five-factor model with the addition of a common method factor could not be fitted in Mplus 8.3 software, which also indicated that there was no serious common method bias.

### 4.2. Confirmatory factor analysis

We conducted confirmatory factor analyses (CFA) of the focal variables using Mplus 8.3. Following the suggestion of Little et al. (2002), we adopt the item parceling techniques before conducting CFA. We parceled self-leadership into three factors and parceled leader empowering behavior into six factors. As Table 1 showed, the four-factor model (*χ*^2^ = 372.216, *df* = 164, *χ*^2^/*df* = 2.270, CFI = 0.917, TLI = 0.904, RMSEA = 0.069, SRMR = 0.058) fit the data better than all other alternative models. Thus, the CFA results showed that the four variables had better discriminative validity.

**Table 1:** Confirmatory factor analyses.

Models	*χ*^2^	*df*	*χ*^2^/*df*	CFI	TLI	RMSEA	SRMR
Four-factor model (SL; AM; JC; LEB)	372.216	164	2.270	0.917	0.904	0.069	0.058
Three-factor model (SL + AM; JC; LEB)	659.477	167	3.949	0.804	0.777	0.105	0.079
Three-factor model (SL; AM + JC; LEB)	790.317	167	4.732	0.751	0.717	0.118	0.119
Three-factor model (SL + LEB; AM; JC)	903.973	167	5.413	0.706	0.666	0.128	0.118
Two-factor model (SL + AM + JC; LEB)	1118.917	169	6.621	0.621	0.574	0.145	0.132
Two-factor model (SL + AM + LEB; JC)	1187.276	169	7.025	0.594	0.544	0.150	0.129
Two-factor model (SL + JC; AM + LEB)	1261.166	169	7.463	0.565	0.510	0.155	0.142
Two-factor model (SL + LEB; AM + JC)	1321.472	169	7.819	0.540	0.483	0.159	0.157
One-factor model (SL + AM+JC + LEB)	1649.355	170	9.702	0.410	0.341	0.180	0.167

*N* = 269. SL, self-leadership; AM, autonomous motivation; JC, job crafting; LEB, leader empowering behavior.

### 4.3. Descriptive statistics and correlations

The means, SDs, and correlations of all variables were presented in Table 2. As shown in Table 2, self-leadership was positively related to job crafting (*r* = 0.313, *p* < 0.001) and autonomous motivation (*r* = 0.377, *p* < 0.001). Hence, Hypothesis 1 and Hypothesis 2 were preliminarily verified. In addition, autonomous motivation was positively related to job crafting (*r* = 0.282, *p* < 0.001), indicating that Hypothesis 3 was initially tested.

**Table 2:** Means, standard deviations, and correlations of variables.

Variable	*M*	SD	1	2	3	4	5	6	7	8
1. Gender	0.540	0.499								
2. Age	2.590	0.731	0.099							
3. Education	3.910	0.770	0.044	0.163^**^						
4. OT	3.060	1.065	−0.001	0.653^***^	0.329^***^					
5. SL	4.307	0.528	0.017	0.083	0.112	0.080	(0.886)			
6. AM	4.208	0.521	0.082	0.242^***^	0.195^**^	0.233^***^	0.377^***^	(0.820)		
7. JC	3.984	0.771	0.045	0.020	0.009	0.033	0.313^***^	0.282^***^	(0.909)	
8. LEB	4.084	0.402	−0.057	−0.036	0.056	−0.002	−0.048	−0.02	0.008	(0.840)

OT, organizational tenure; SL, self-leadership; AM, autonomous motivation; JC, job crafting; LEB, leader empowering behavior. Values on the diagonal are the Cronbach’s alpha coefficient of each scale. ^*^p < 0.05, ^**^p < 0.01, ^***^p < 0.001.

### 4.4. Hypothesis testing

To test the research hypotheses, we conducted hierarchical regression analysis using SPSS 26 software. Table 3 showed the detailed results of hierarchical regression analysis. To start with, Model 2 revealed that after controlling for the effects of all control variables, self-leadership had a significantly positive effect on autonomous motivation (*β* = 0.346, *p* < 0.001), indicating that Hypothesis 2 was supported. Similarly, we introduced all control variables into Model 4 and then added self-leadership into Model 5. Model 5 showed that after controlling for the effects of all control variables, self-leadership had a significantly positive effect on job crafting (*β* = 0.315, *p* < 0.001). Thus, Hypothesis 1 was verified. Besides, Model 6 indicated that after controlling demographic variables, autonomous motivation had a significantly positive effect on job crafting (*β* = 0.299, *p* < 0.001). Accordingly, Hypothesis 3 was supported. Then, comparing Model 5 and Model 7, after introducing autonomous motivation, the coefficient of the effect of self-leadership on job crafting decreased (*β* = 0.315, *p* < 0.001, Model 5; *β* = 0.243, *p* < 0.001, Model 7). Meanwhile, autonomous motivation still had a significantly positive impact on job crafting (*β* = 0.208, *p* < 0.01). Thus, it can be found that autonomous motivation played a partial mediating role between self-leadership and job crafting. Whereby, Hypothesis 4 was supported. In addition, we also utilized Model 4 in the PROCESS macro of Hayes (2013) to examine the mediating effect of autonomous motivation. Results from 5,000 times bootstrapping indicated that the indirect effect of self-leadership on job crafting *via* autonomous motivation was significant (indirect effect = 0.105, 95% CI = [0.039,0.177], excluding 0). Therefore, Hypothesis 4 was further verified.

**Table 3:** Results of hierarchical regression analyses.

Variable	Autonomous motivation			Job crafting			
	Model 1	Model 2	Model 3	Model 4	Model 5	Model 6	Model 7
Gender	0.060	0.058	0.054	0.047	0.045	0.029	0.033
Age	0.158^*^	0.137	0.141	−0.010	−0.029	−0.057	−0.058
Education	0.138^*^	0.104	0.122^*^	−0.005	−0.036	−0.046	−0.058
OT	0.085	0.082	0.079	0.041	−0.039	0.016	0.022
SL		0.346^***^	0.342^***^		0.315^***^		0.243^***^
LEB			0.011				
SL*LEB			0.122^*^				
AM						0.299^***^	0.208^**^
*F*	6.513^***^	13.788^***^	10.640^***^	0.213	5.910^***^	4.852^***^	6.826^***^
*R*^2^	0.090	0.208	0.222	0.003	0.101	0.084	0.135
Adjusted *R*^2^	0.076	0.193	0.201	−0.012	0.084	0.067	0.115
△*R*^2^		0.118	0.014		0.098	0.081	0.034

OT, organizational tenure; SL, self-leadership; AM, autonomous motivation; JC, job crafting; LEB, leader empowering behavior. All regression coefficients are standard regression coefficients. ^*^p < 0.05, ^**^p < 0.01, ^***^p < 0.001.

**Figure 2:**
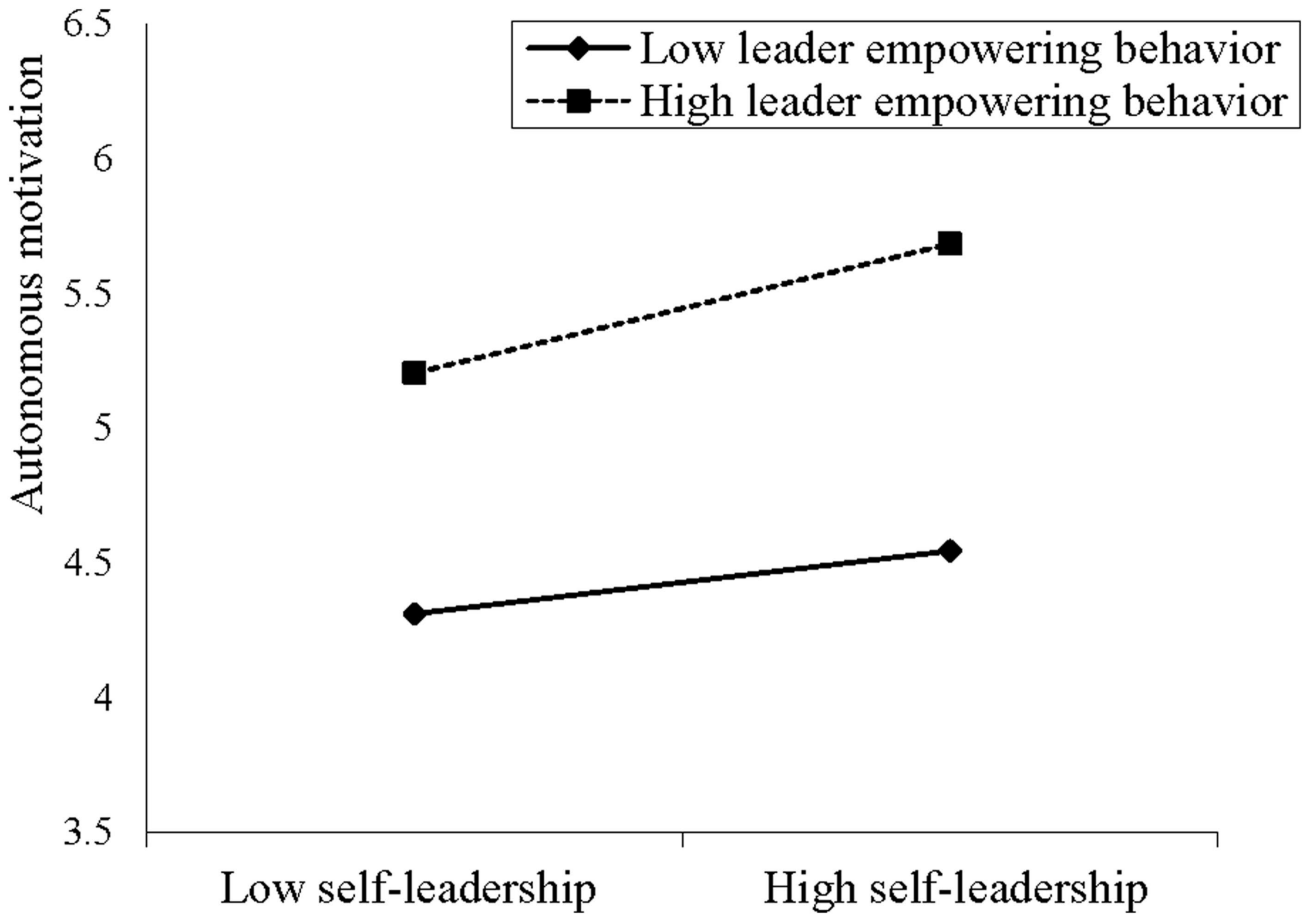
Moderating effect of leader empowering behavior on relationship between self-leadership and autonomous motivation.

We also utilized a three-step hierarchical regression analysis to verify the moderating role of leader empowering behavior. To reduce multi-collinearity problems, the independent variable and moderator variable were centralized in the light of Aiken and West’s (1991) instructions. As shown in Table 3, Model 3 showed that the interaction coefficient of self-leadership and leader empowering behavior was significantly and positively related to autonomous motivation (*β* = 0.122, *p* < 0.05), demonstrating that leader empowering behavior played a positive moderating role in the relationship between self-leadership and autonomous motivation. Thus, Hypothesis 5 was supported. In order to further verify the moderating effect, we also performed the simple slope test and plotted the moderating effects, as shown in Figure 2. The results indicated that under the high level of leader empowering behavior, the positive relationship between self-leadership and autonomous motivation was stronger (effect = 0.455, SE = 0.075, *p* < 0.001, 95% CI = [0.307, 0.603], excluding 0). Conversely, under the low level of leader empowering behavior, the positive relationship between self-leadership and autonomous motivation was weaker (effect = 0.221, SE = 0.077, *p* < 0.01, 95% CI = [0.069, 0.374], excluding 0). These results further corroborated Hypothesis 5.

Finally, using Model 7 in the PROCESS macro, this study calculated the mediating effects of self-leadership on job crafting *via* autonomous motivation at three values of leader empowering behavior to verify the moderated mediation effect. The detailed results were presented in Table 4. Based on 5,000 bootstrapping samples, the results revealed that under the low level of leader empowering behavior, the indirect effect was lower (indirect effect = 0.068, 95% confidence interval = [0.011, 0.140], excluding 0). In contrast, under the high level of leader empowering behavior, the indirect effect was higher (indirect effect = 0.140, 95% confidence interval = [0.051, 0.234], excluding 0). Moreover, results indicated that the index of moderated mediation was significant (indirect effect = 0.089, 95% confidence interval = [0.013, 0.197], excluding 0). Therefore, leader empowering behavior significantly and positively moderates the mediating role of autonomous motivation, supporting Hypothesis 6.

**Table 4:** Results of the moderated mediation.

Leader empowering behavior	Self-leadership → autonomous motivation → job crafting		
	Indirect effect	S.E.	95% CI
Low (mean − 1SD)	0.068	0.033	[0.011, 0.140]
Mean	0.104	0.035	[0.037, 0.177]
High (mean + 1SD)	0.140	0.046	[0.051, 0.234]

## 5. Discussion

According to self-determination theory, the present study established a moderated mediation model to reveal the internal mechanism and boundary conditions on the impact of self-leadership on employees’ job crafting. Using 269 three-wave employee samples, we found that self-leadership had a significantly positive effect on employees’ job crafting and that autonomous motivation played a mediating effect in the positive relationship between self-leadership and job crafting. Besides, leader empowering behavior positively moderated not only the relationship between self-leadership and autonomous motivation, but also the mediating effect of autonomous motivation between self-leadership and job crafting. These findings contributed to the literature and provided insights into management practice.

### 5.1. Theoretical implications

Our study makes several theoretical contributions in the following ways. First, we enrich the literature on job crafting by identifying self-leadership as a vital antecedent variable of employees’ job crafting. Existing researches mainly focus on the positive outcomes (e.g., job satisfaction, Villajos et al., 2019; job performance, Bakker et al., 2012) and antecedents related to job characteristics (e.g., task significance, Zhang and Parker, 2019; task interdependence, Niessen et al., 2016) of job crafting. Meanwhile, a few studies have begun to explore the impact of different leadership styles and leadership behaviors (e.g., transformational leadership, Wang et al., 2017) on job crafting from a top-down perspective, however, few studies have explored the influence of employees’ self-oriented leadership styles on job crafting from a bottom-up perspective. Different from the typical leadership styles, self-leadership is a process of leading from the inside out and specifies an individual as both the initiator and the target of influence (Knotts et al., 2022). Therefore, this study enriches our understanding of the antecedents of employees’ job crafting from a new perspective and reveals the key function of self-leadership in the process of promoting employees’ job crafting.

Second, based on self-determination theory, we make contributions to the literature by revealing the underlying mechanism in the relationship between self-leadership and job crafting. Our findings indicated that autonomous motivation had a mediating effect on the influence of self-leadership on job crafting, which provided a new internal mechanism for the formation of job crafting and broadened the application scope of self-determination theory. Moreover, Zhang and Parker (2019) have proposed that previous scholars have not fully clarified the intrinsic autonomous motivation of employees’ job crafting. This study responded to their argument by exploring the mediating effect of autonomous motivation. Therefore, this study not only helps to explain the key question of “how” self-leadership influences employees’ job crafting from the perspective of motivation, but also expands the research on the formation mechanism of job crafting.

Finally, our study investigates and examines the moderating role of leader empowering behavior and reveals the boundary condition under which self-leadership affects employees’ job crafting *via* autonomous motivation. In regard to the boundaries of self-leadership, previous studies primarily focused on cultural context (e.g., power distance; Harari et al., 2021) and job characteristics (e.g., job autonomy; Ho and Nesbit, 2014), but paid less attention to leadership behaviors. Combining self-determination theory and the self-leadership model, this study found that leader empowering behavior could enhance the positive effect of self-leadership on autonomous motivation, and further promote job crafting. The study result reveals that positive external contextual factors can promote the effective play of self-leadership, and verifies that the external resources support of the organization contributes to the sustainable operation of self-leadership from the perspective of resources (Stewart et al., 2019). Therefore, this study answers the question of “when” self-leadership promotes employees’ job crafting, and enriches the understanding of relevant contextual factors that enhance the effects of self-leadership.

### 5.2. Managerial implications

Our study also has several significant managerial implications. First of all, the findings show that self-leadership has a positive impact on employees’ job crafting. Thus, an effective measure to promote employees’ job crafting is for organizations to take appropriate measures to encourage them to adopt self-leadership. For example, organizations should create the necessary conditions and conduct self-leadership training programs for employees, which can help employees to master better self-leadership strategies, such as self-goal setting, self-observation, and self-reward, and improve self-leadership skills. Moreover, in the recruitment process, the organization can measure self-leadership and select employees with high self-leadership.

Second, the findings show that self-leadership positively affects employees’ job crafting through the mediating role of autonomous motivation. In other words, when employees have strong autonomous motivation, they will engage in more job crafting. Therefore, organizations should take effective measures to promote the internalization of external motivation and improve the autonomous motivation of employees, thereby providing an internal force for employees to carry out job crafting. On the one hand, the organization should create a supportive environment and harmonious work atmosphere, and establish a two-way communication feedback mechanism to meet employees’ psychological needs for autonomy. On the other hand, the organization should take actions to cultivate the abilities of employees, such as skill training and work shift, and reduce the pressure on employees, so as to meet their psychological needs for competence. Furthermore, managers should express appreciation and gratitude to employees, especially the employees who perform essential tasks well, thus establishing good supervisor-subordinate guanxi and meeting their psychological needs for relatedness.

Finally, our results indicate that leader empowering behavior plays a moderating role in the positive impact of self-leadership on job crafting *via* autonomous motivation. Therefore, leaders should pay attention to engaging in more empowering behaviors to strengthen the positive influence of self-leadership. For example, organization leaders can encourage employees to engage in decision-making by proactively soliciting their ideas and suggestions, give them more autonomy by allowing them to accomplish work tasks in their own ways, and ensure the sustainable operation of employee self-leadership by providing adequate resources. In addition, the organization should establish a transparent information sharing mechanism, maintain the flexibility of the organization, and enhance the employees’ self-control and self-confidence, so as to enhance the interaction between self-leadership and autonomous motivation, and further promote employees’ job crafting.

### 5.3. Limitations and future research

Even though our study contributes to theory and practice, there still are some shortcomings that could be improved in future studies. First, although this study performed a three-wave survey with a two-week time lag to obtain the data, each variable was reported by a single source, making it difficult to avoid common method bias (Podsakoff et al., 2003) and unambiguously establish causality. Therefore, future studies could conduct a longitudinal or experimental design to further verify the validity of the research results. In addition, since the data was solely obtained in the Chinese context, it is doubtful whether the results of our study can be generalized to other cultures. For instance, in the cultural context of low power distance, employees may have different levels of self-leadership and the impact of self-leadership will be different (Ho and Nesbit, 2014). Thus, future scholars are recommended to test the theoretical model in different cultures to extend the research results.

Second, this study only revealed the mediating role of autonomous motivation based on self-determination theory. The finding showed that autonomous motivation played a partial mediating role between self-leadership and employees’ job crafting, indicating there may be other underlying mechanisms. Thus, future researchers can further explore the potential mediating variables. Besides, job crafting, as a self-oriented proactive behavior (Wrzesniewski and Dutton, 2001), is often closely related to other abilities, motivations, and opportunities. Therefore, based on the ability-motivation-opportunity model, future studies can further explore how to promote employees’ job crafting by improving crafting ability, stimulating crafting motivation, and providing crafting opportunities.

Finally, this study only investigated the moderating role of leader empowering behavior in the relationship between self-leadership and autonomous motivation. Based on the self-leadership model, external resources support and the internal forces of employees help to provide sufficient resource supply to ensure the normal progress of the self-leadership process (Stewart et al., 2019). Combined with self-determination theory, when self-leadership effectively interacts with external contextual factors or internal forces, it will have a more positive influence on employees’ attitudes and behaviors (Ryan and Deci, 2000). Therefore, future research can explore the moderating effects of individual internal factors, such as harmonious passion.

## 6. Conclusion

We investigated the underlying mechanism of the relationship between self-leadership and employees’ job crafting and the moderating role of leader empowering behavior. The findings deepened our understanding of how and when self-leadership affected employees’ job crafting from the perspective of self-determination theory. Specifically, self-leadership had a significant positive effect on employees’ autonomous motivation and job crafting. Autonomous motivation partly mediated the positive relationship between self-leadership and job crafting. Moreover, leader empowering behavior moderated the positive influence of self-leadership on autonomous motivation and the mediating role of autonomous motivation between self-leadership and job crafting.

## Data availability statement

The raw data supporting the conclusions of this article will be made available by the authors, without undue reservation.

## Ethics statement

All procedures were conducted in compliance with the ethics code and approved by the authors’ department. Written informed consent for participation was not required for this study in accordance with the national legislation and the institutional require.

## Author contributions

GL mainly designed the research, analyzed data, as well as wrote and revised the paper. HP mainly led literature review and data collection. HW mainly collected data and revised the manuscript. All authors contributed to the article and approved the submitted version.

## Funding

This research was funded by Science and Technology Research Project of Education Department of Jiangxi Province (GJJ2200403).

## Conflict of interest

The authors declare that the research was conducted in the absence of any commercial or financial relationships that could be construed as a potential conflict of interest.

## Publisher’s note

All claims expressed in this article are solely those of the authors and do not necessarily represent those of their affiliated organizations, or those of the publisher, the editors and the reviewers. Any product that may be evaluated in this article, or claim that may be made by its manufacturer, is not guaranteed or endorsed by the publisher.
